# Differential regulation of microRNA-146a and microRNA-146b-5p in human retinal pigment epithelial cells by interleukin-1β, tumor necrosis factor-α, and interferon-γ

**Published:** 2013-04-03

**Authors:** R. Krishnan Kutty, Chandrasekharam N. Nagineni, William Samuel, Camasamudram Vijayasarathy, Cynthia Jaworski, Todd Duncan, Jennifer E. Cameron, Erik K. Flemington, John J. Hooks, T. Michael Redmond

**Affiliations:** 1 Laboratory of Retinal Cell and Molecular Biology, National Eye Institute, National Institutes of Health, Bethesda, MD; 2 Laboratory of Immunology, National Eye Institute, National Institutes of Health, Bethesda, MD; 3 National Institute on Deafness and Other Communication Disorders, National Institutes of Health, Bethesda, MD; 4 Tulane Cancer Center, Tulane University, New Orleans, LA; 5 Microbiology, Immunology & Parasitology and the Stanley S. Scott Cancer Center, Louisiana State University Health Sciences Center, New Orleans, LA

## Abstract

**Purpose:**

The inflammatory response of the retinal pigment epithelium (RPE) is implicated in the pathogenesis of age-related macular degeneration. The microRNAs miR-146a and miR-146b-5p can regulate the inflammatory process by attenuating cytokine signaling via the nuclear factor-κB pathway. The aim of the present study is to investigate the expression of miR-146a and miR-146b-5p in human RPE cells and their response to proinflammatory cytokines.

**Methods:**

Confluent cultures of RPE cells established from adult human donor eyes were treated with the proinflammatory cytokines interferon (IFN)-γ, tumor necrosis factor (TNF)-α, and interleukin (IL)-1β. The expression of microRNAs was analyzed by real-time PCR using total RNA fraction. The retinal pigment epithelial cell line ARPE-19 was employed to analyze the promoter activity of the genes encoding miR-146a and miR-146b-5p. STAT1-binding activity of oligonucleotides was analyzed by electrophoretic mobility shift assay. ARPE-19 cells were transiently transfected with miR-146a and miR-146b-5p mimics for the analysis of IRAK1 expression by western immunoblotting.

**Results:**

Real-time PCR analysis showed that miR-146a and 146b-5p are expressed in RPE cells. The cells responded to proinflammatory cytokines (IFN-γ + TNF-α + IL-1β) by highly increasing the expression of both miR-146a and miR-146b-5p. This was associated with an increase in the expression of transcripts for *CCL2*, *CCL5*, *CXCL9*, *CXCL10*, and *IL-6*, and a decrease in that for *HMOX1*. The miR-146a induction was more dependent on IL-1β, since its omission from the cytokine mix resulted in a greatly reduced response. Similarly, the induction of miR-146b-5p was more dependent on IFN-γ, since its omission from the cytokine mix minimized the effect. In addition, the increase in *MIR146B* promoter activity by the cytokine mix was effectively blocked by JAK inhibitor 1, a known inhibitor of the JAK/STAT signaling pathway. The expression of IRAK1 protein was decreased when ARPE-19 cells were transiently transfected with either miR-146a mimic or miR-146b-5p mimic.

**Conclusions:**

Our results clearly show that both miR-146a and miR-146b-5p are expressed in human RPE cells in culture and their expression is highly induced by proinflammatory cytokines (IFN-γ + TNF-α + IL-1β). The induction of miR-146a showed a dependency on IL-1β, while that of miR-146b-5p on IFN-γ. Our results show for the first time that miR-146b-5p expression is regulated by IFN-γ, potentially via the JAK/STAT pathway. These two microRNAs could play a role in inflammatory processes underlying age-related macular degeneration or other retinal degenerative diseases through their ability to negatively regulate the nuclear factor-κB pathway by targeting the expression of IRAK1.

## Introduction

A normally functioning retinal pigment epithelium (RPE) is indispensable for vision. It also maintains the immune privilege of the retina by serving as a blood/retina barrier and by secreting immunosuppressive factors [1]. Ocular inflammation is often associated with the infiltration of lymphocytes and macrophages to the posterior compartment of the eye and their secretion of inflammatory mediators such as interferon (IFN)-γ, tumor necrosis factor (TNF)-α, and interleukin (IL)-1β [2,3]. These proinflammatory cytokines can target the RPE and trigger inflammatory responses. The loss of critical RPE functions resulting from uncontrolled inflammatory response could be an important factor in the pathogenesis of age-related macular degeneration (AMD) and other retinal degenerative disorders [4-6]. Human RPE (HRPE) cells in culture do respond to IFN-γ, TNF-α, and IL-1β by increasing the expression of cytokines and chemokines [7-14].

MicroRNAs (miRNAs), single-stranded noncoding small (~22 nucleotides) RNA molecules, control many eukaryotic cellular functions by regulating gene expression postranscriptionally [15,16]. In humans, miRNAs are encoded by over 1,600 genes localized to different chromosomes. They are initially transcribed as primary transcripts (pri-miRNAs) before being processed to pre-miRNAs and finally to mature miRNAs. A mature miRNA, an essential component of RNA-initiated silencing complex, can bind and target gene transcripts for destabilization or translational repression. A perfect complementarity between the miRNA and its target messenger RNA often results in destabilization of the latter by rapid degradation. Binding of the miRNA to the 3′-untranslated region inhibits the translation of the target messenger RNA. The translational repression requires only a partial complementarity between the miRNA and its target transcripts. Posttranscriptional gene silencing by two closely related microRNAs, miR-146a and miR-146b-5p (also known as miR-146b), is known to play important role in regulating inflammatory response. The expression of miR-146a and miR-146b-5p are greatly increased in human monocytes by lipopolysaccharide, TNF-α, and IL-1β [17]. Mature forms of miR-146a and miR-146b-5p are encoded by two separate genes—*MIR146A* and *MIR146B*—localized to human chromosomes 5 and 10, respectively [17]. They have similar sequences except for two bases toward the 3′-end, and therefore could target the same transcript for translational repression. These miRNAs function as negative regulators of inflammatory process due to their ability to target interleukin-1-receptor-associated kinase-1 (IRAK1) and TNF receptor associated factor 6 (TRAF6), known modulators of nuclear factor-kappaB (NF-κB) pathway, for translational repression and thereby inhibiting proinflammatory cytokine signaling [17-19]. Excessive inflammatory response exhibited by miR-146a knockout mouse clearly supports the role of this microRNA as a negative modulator of inflammatory response [20]. In addition, alteration in the expression of miR-146a and/or miR-146b-5p has been reported to be associated with infection and inflammatory diseases [17,21-27].

The potential role of miR-146a and miR-146b-5p in regulating the inflammatory response of HRPE is not yet known. Therefore, we investigated whether these miRNAs are expressed in RPE cells and how they respond to proinflammatory cytokines TNF-α, IL-1β, and IFN-γ. Here, we show that both miR-146a and miR-146b-5p are indeed expressed in HRPE cells in culture and their expression is highly increased in these cells when exposed to proinflammatory cytokines.

## Methods

### Cell culture

HRPE cell cultures were established from eyes of normal adult human donors of ages 77, 81, and 87 [10]. The cells (passages 7 to 11) were grown to confluence in 100 mm dishes or 6 well plates using minimum essential medium supplemented with 10% fetal bovine serum (FBS), nonessential amino acids, and antibiotic-antimycotic mixture at 37 °C in a humidified environment of 5% CO_2_ in air [10]. Reagents for cell culture including media and FBS were purchased from Invitrogen (Carlsbad, CA). The HRPE cells used in these studies retained typical epithelial morphology from passages 7 through 11 as evident from the polygonal and cuboidal appearance of the cells with clear intercellular junctions during the examination with an inverted microscope, and also from positive immunostaining of all the cells by an antibody against cytokeratin.

The ARPE-19 human retinal pigment epithelial cell line was obtained from ATCC (Manassas, VA). The cells (passages 23 through 26) were grown in Dulbecco’s modified Eagle’s medium containing nutrient mixture F12, 50/50 mix (Cellgro, Herndon, VA) supplemented with 5% FBS, 2 mM L-glutamine, 1 mM sodium pyruvate, 0.1 mM nonessential amino acids, penicillin (100 U/ml), and streptomycin (100 μg/ml), as described previously [28].

Human recombinant TNF-α and IFN-γ were purchased from Roche Applied Science (Indianapolis, IN) and IL-1β was from R&D Systems (Minneapolis, MN). The confluent cell cultures were treated with the inflammatory cytokines (TNF-α, 10 ng/ml; IL-1β, 10 ng/ml; and IFN-γ, 100 u/ml) in the absence of serum for 16 h unless otherwise indicated. The cells were viable and did not show any sign of apoptosis when tested for DNA fragmentation following the treatment.

### Real-Time PCR

The total RNA fraction containing miRNAs was prepared from control or treated cells using Ambion mirVana miRNA isolation kit (Applied Biosystems, Foster City, CA) and the expression of miRNAs was analyzed by real-time PCR as described before [29]. Briefly, the RNA preparation was reverse transcribed and then analyzed by real-time PCR using predesigned primers and TaqMan probes specific for the target miRNA following manufacturer’s instructions. Individual TaqMan MicroRNA Assays (hsa-miR-146a and hsa-miR-146b-5p, hsa-miR-125b, hsa-miR-155, hsa-miR-181d, hsa-miR-204, hsa-miR-218, hsa-miR-30b, hsa-miR-455–3p, hsa-miR-638, and hsa-miR-7), TaqMan MicroRNA Reverse Transcription Kit and TaqMan Universal PCR Master Mix, No AmpErase UNG, and the endogenous control RNU48 were obtained from Applied Biosystems. Applied Biosystems Real-Time PCR Systems (7500 or 7900HT) were employed for all real-time PCR analysis, following the manufacturer’s default thermal cycling conditions.

For quantitative real-time reverse transcriptase polymerase chain reaction (RT–PCR) analysis of various transcripts, 2 µg of total RNA was reverse transcribed using a High Capacity cDNA Archive Kit (Applied Biosystems). After reverse transcription, the cDNA was used as templates for quantitative real-time PCR using TaqMan Universal PCR Master Mix and other reagents from Applied Biosystems. Each PCR reaction (20 µl) was set up using validated TaqMan probes (labeled with reporter dye FAM at the 5′ end) and primers specific for each gene (*CCL2*, *CCL5*, *CXCL9*, *CXCL10*, *IL-6*, and *HMOX1*) with assay identification numbers Hs00234140_m1, Hs99999048_m1, Hs00171065_m1, Hs99999049_m1, Hs99999032_m1, and Hs00157965_m1, respectively). Human *GAPDH* (part number: 4352934E) gene was used as the endogenous control. Gene amplification data were analyzed with an Applied Biosystems 7500 System Sequence Detection Software version 1.2.3. The results were expressed as n-fold induction in gene expression calculated using the relative quantification (ΔΔCT) method.

### Electrophoretic mobility shift assay

Confluent cultures of HRPE cells were treated with IFN-γ (100 u/ml) or cytokine mixture (TNF-α, 10 ng/ml; IL-1β, 10 ng/ml; and IFN-γ, 100 u/ml) for 6 h. Nuclear extracts were prepared from control and treated cells according to the manufacturer’s instructions (Active Motif, Carlsbad, CA). Electrophoretic mobility shift assays were performed using the LightShift chemiluminescent electrophoretic mobility shift assay kit (Pierce, Rockford, IL). The probes were prepared by annealing complimentary oligonucleotides labeled with biotin at the 5′-end. The biotin-labeled oligonucleotides were purchased from Integrated DNA Technologies (Coralville, IA). The oligonucleotide containing the putative STAT1 binding element present in the miR-146b-5p promoter region has the forward sequence of 5′-CCT TCC TCC TTT CTC AGA AGA GCC AGC-3′. The oligonucleotide used as a positive control for STAT1 binding had the forward sequence of 5′-GTT ATT TCC CAG AAA GGC CAG ACA T-3′. The DNA-protein binding was performed for 20 min at room temperature in a final volume of 20 µl containing 1X binding buffer (10 mM Tris, pH 7.5, 1 mM DTT, 50 mM KCl), 5% glycerol (v/v), 5 mM MgCl_2_, 0.05% NP-40, 0.05 µg poly(dI-dC), 50 fmol double-stranded biotinylated probe, and 2 µg nuclear extract. For the competition assay, 100X concentrated unlabeled probe was included in the binding reaction. The protein/DNA complexes were separated on 6% nondenaturing polyacrylamide gel at 100 V using 0.5X TBE buffer (45 mM tris(hydroxymethyl)aminomethane, 45 mM boric acid and 1 mM ethylenediamine tetraacetic acid; pH 8.3). The biotin labeled DNA–protein complexes in the gel were transferred to Hybond-N+ nylon membrane (GE Health Care, Pittsburgh, PA) and ultraviolet crosslinked to the membrane. The shifted bands corresponding to the protein/DNA complexes relative to the unbound double-stranded DNA were visualized by exposing the membrane to a film after sequentially treating it with streptavidin–horseradish peroxidase conjugate and chemiluminescent substrate.

### Promoter activity

The cloning of the promoter region of *MIR146A* (chr 5:159,894,126–159,895,278 bp) or that of *MIR146B* (chr 10:104,194,716–104,195,728 bp) into pGL3 basic vector (Promega, Madison, WI) to yield the promoter/luciferase reporter constructs miR-146a pro 869–2021 pGL3basic wt and miR-146b pro 1148–2160 pGL3basic, respectively, has been described [22] (constructs were obtained from Flemington lab). ARPE-19 cells were plated in six-well culture dishes at a density of 1x10^5^ cells/well and maintained at 37 ^°^C in an atmosphere of 5% CO_2_ overnight, and transfection was performed using X-treme gene HP DNA transfection reagent (Roche) according to the manufacturer’s recommendations. Briefly, 2 μg of promoter–firefly luciferase reporter construct and 20 ng of Renilla luciferase vector (pRL-TK) were mixed with 2 μl of transfection reagent in 200 μl of OptiMem® I Medium (Life Technologies, Grand Island, NY). The transfection reagent:DNA complex was incubated for 15 min at 25 °C, then mixed with 2 ml of complete culture medium and used to replace culture medium in each well of the six-well culture dish. The transfection was allowed to continue for 24 h by incubating culture dishes at 37 °C. The cell culture medium was removed from each well and the cells were briefly washed with serum-free medium before treating the cells with indicated combinations of TNF-α (10 ng/ml), IL-1β (10 ng/ml), and IFN-γ (100 u/ml) in 2 ml serum-free medium for another 24 h at 37 °C. Cells were preincubated with 0.1 or 0.5 μM of JAK inhibitor-1 (Calbiochem, San Diego, CA) for 1 h before the cytokine treatment, when necessary. The cells were lysed in 250 μl of 1X Passive Lysis Buffer and stored at −20 °C until assayed. The luciferase activity was measured using a Dual Luciferase Reporter Assay System (Promega) according to the manufacturer’s instructions, and was expressed as relative luciferase activity by normalizing firefly luciferase activity against *Renilla* luciferase activity.

### Transfection with microRNA mimics and western immunoblot analysis

The miScript miRNA mimics for miR-146a, miR-146b-5p, and negative control, as well as HiPerfect transfection reagent were purchased from Qiagen Inc. (Valencia, CA). ARPE-19 cells (1.86×10^6^ cells in 100 mm culture dish) were transiently transfected with miRNA mimics using the protocol supplied by the manufacturer. The cells were harvested after 72 h by trypsin treatment. The cells were suspended in Cell Lysis Buffer (Cell Signaling Technology, Danvers, MA) at 4 °C, sonicated, and then centrifuged at 12,000× *g* for 10 min. Equal amounts of the supernatants (25 µg protein) were subjected to sodium dodecyl sulfate–polyacrylamide gel electrophoresis and then blotted on to a Hybond-N^+^ nylon membrane. The blot was then probed for IRAK1 using mouse anti-IRAK1 monoclonal antibody (1:100 dilution, Santa Cruz) and IRDye 800CW goat antimouse IgG. The blot was then stripped and reprobed with mouse anti-actin monoclonal antibody (Sigma-Aldrich, St. Louis, MO) and IRDye 680LT goat antimouse IgG. The blocking buffer and the IRDye-labeled secondary antibodies were purchased from Li-Cor Biotechnology (Lincoln, NE). The immunoreactive bands on the blots were detected using a Li-Cor Odyssey Clx Infrared Imaging System.

### Statistical analysis

A paired Student *t* test was used for the analysis of statistical significance. A p value less than or equal to 0.05 was considered statistically significant. Representative of replicate experiments are shown in the figures, and values are shown as mean ± standard deviation.

## Results

**Figure 1:**
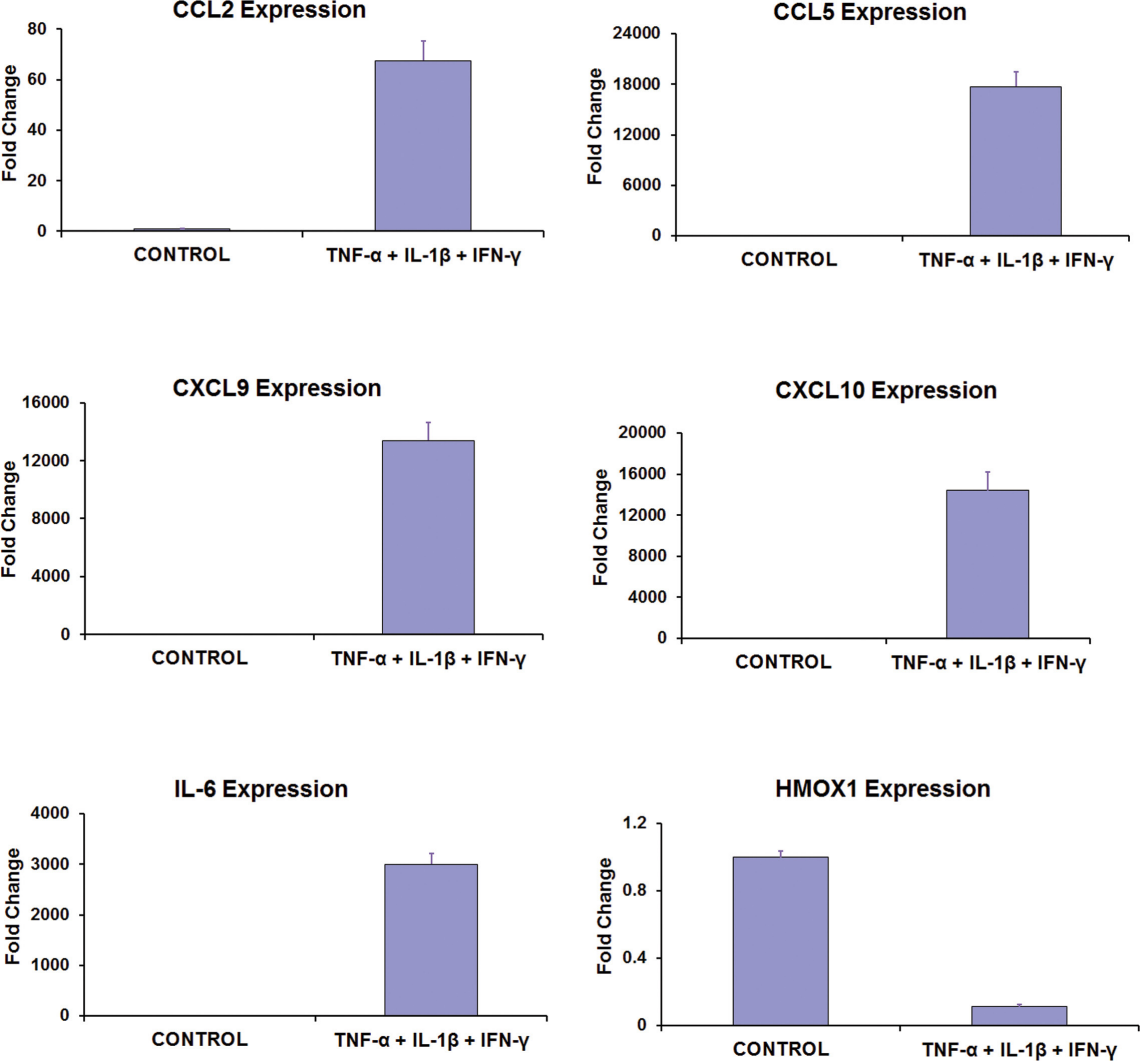
Human retinal pigment epithelial cells responded to proinflammatory cytokines by markedly altering the expression of several genes. The cells were treated with a cytokine mixture consisting of interferon (IFN)-γ (100 u/ml), tumor necrosis factor (TNF)-α (10 ng/ml), and interleukin (IL)-1β (10 ng/ml) for 16 h. Total RNA fractions isolated from control and treated cells were reverse-transcribed and the gene expression analyzed by real-time PCR using the relative quantification method. The mean C_T_ values were as follows: control=26.95±0.02 and treated=19.84±0.04 for CCL2; control=33.04±0.08 and treated=17.96±0.02 for CCL5; control=32.89±0.09 and treated=18.41±0.04 for CXCL9; control=31.95±0.02 and treated=17.23±0.02 for CXCL10; control=30.93±0.02 and treated=18.79±0.01 for IL-6; control=24.95±0.02 and treated=27.45±0.02 for HMOX1; and control=19.53±0.07 and treated=18.94±0.03 for GAPDH (endogenous control). Expression of CCL2, CCL5, CXCL9, CXCL10, and IL-6 was increased and that of HMOX1 decreased following the treatment; p<0.001, n=3. The data shown are representative of three different experiments.

**Figure 2:**
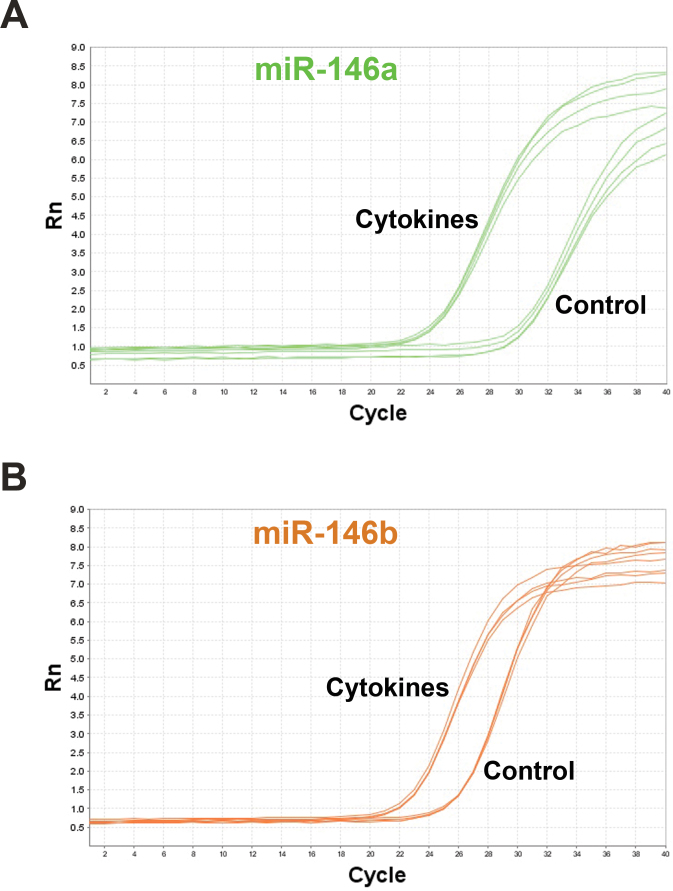
Proinflammatory cytokines increased the expression of miR-146a and miR-146b-5p in human retinal pigment epithelial cells. The cells were treated with interferon (IFN)-γ (100 u/ml), tumor necrosis factor (TNF)-α (10 ng/ml), and interleukin (IL)-1β (10 ng/ml) for 16 h and the microRNA (miRNA) expression was analyzed by real-time PCR. The amplification plots generated shows that the expression of both miR-146a and miR-146b-5p is increased when the cells are exposed to inflammatory cytokines. The data from one of four similar experiments are shown here.

HRPE cells in culture are known to respond to IFN-γ, TNF-α, and IL-1β by increasing the expression of cytokines and chemokines [7-10,30]. The expression of miR-146a and miR-146b-5p was analyzed in HRPE cell cultures under these conditions. The cells were first treated with a mixture of IFN-γ, TNF-α, and IL-1β for 16 h, as described previously [10,29,30], and effectiveness of the treatment was assessed by analyzing the expression of several genes known to be regulated by these cytokines. Real-time PCR analysis showed that the cytokine treatment highly increased the expression of *CCL2*, *CCL5*, *CXCL9*, *CXCL10*, and *IL-6*, while substantially decreasing the expression of *HMOX1*, as expected (Figure 1). Total RNA fractions extracted from control and treated cells were reverse transcribed and the expression of mature forms of miR-146a and miR-146b-5p was analyzed by real-time PCR. Both these miRNAs were expressed in RPE cells as indicated by the PCR amplification plots (Figure 2). It appears that miR-146b-5p could be expressed more abundantly in control cells compared to miR-146a based on the lower cycle threshold (Ct) value for the former. The proinflammatory cytokines greatly induced the expression of both miR-146a and miR-146b-5p in HRPE cells. However, the magnitude of induction appears to be considerably higher for miR-146a in comparison to miR-146b-5p.

**Figure 3:**
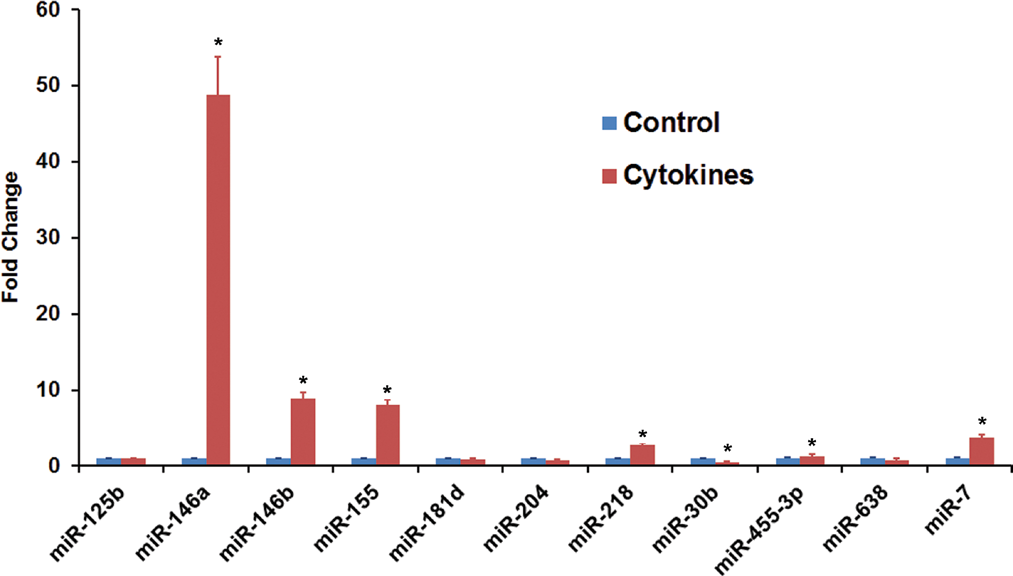
Reverse-transcriptase polymerase chain reaction analysis shows that the microRNA expression in human retinal pigment epithelial cells is altered by proinflammatory cytokines. The cells were treated with interferon (IFN)-γ (100 u/ml), tumor necrosis factor (TNF)-α (10 ng/ml), and interleukin (IL)-1β (10 ng/ml) for 16 h. Total RNA fractions were obtained from control or treated cells and the expression of selected microRNAs (miRNAs) were analyzed by real-time PCR. Increase in the expression in response to the proinflammatory cytokines was more pronounced in the case of miR-146a, miR-146b-5p, and miR-155. *p<0.0001 when compared to respective control, n=4. The data shown are representative of three separate experiments.

We have reported previously that the expression of miR-155 in HRPE cells is regulated by the proinflammatory cytokines [29]. Therefore, the response of miR-146a and miR-146b-5p to the cytokines was compared to that of miR-155 by real-time PCR analysis (Figure 3). We also included eight other miRNAs known to be expressed in HRPE cells [29]. The cytokine treatment increased the expression of miR-146a and miR-146b-5p by up to 50 and eightfold, respectively. In comparison, the expression of miR-155 was increased by ~8-fold, as expected. The expression of miR-218, miR-455–3p and miR-7 was increased to a lesser extent and that of miR-30b was decreased. The remainder of the miRNAs showed no significant changes in response to the cytokine treatment.

**Figure 4:**
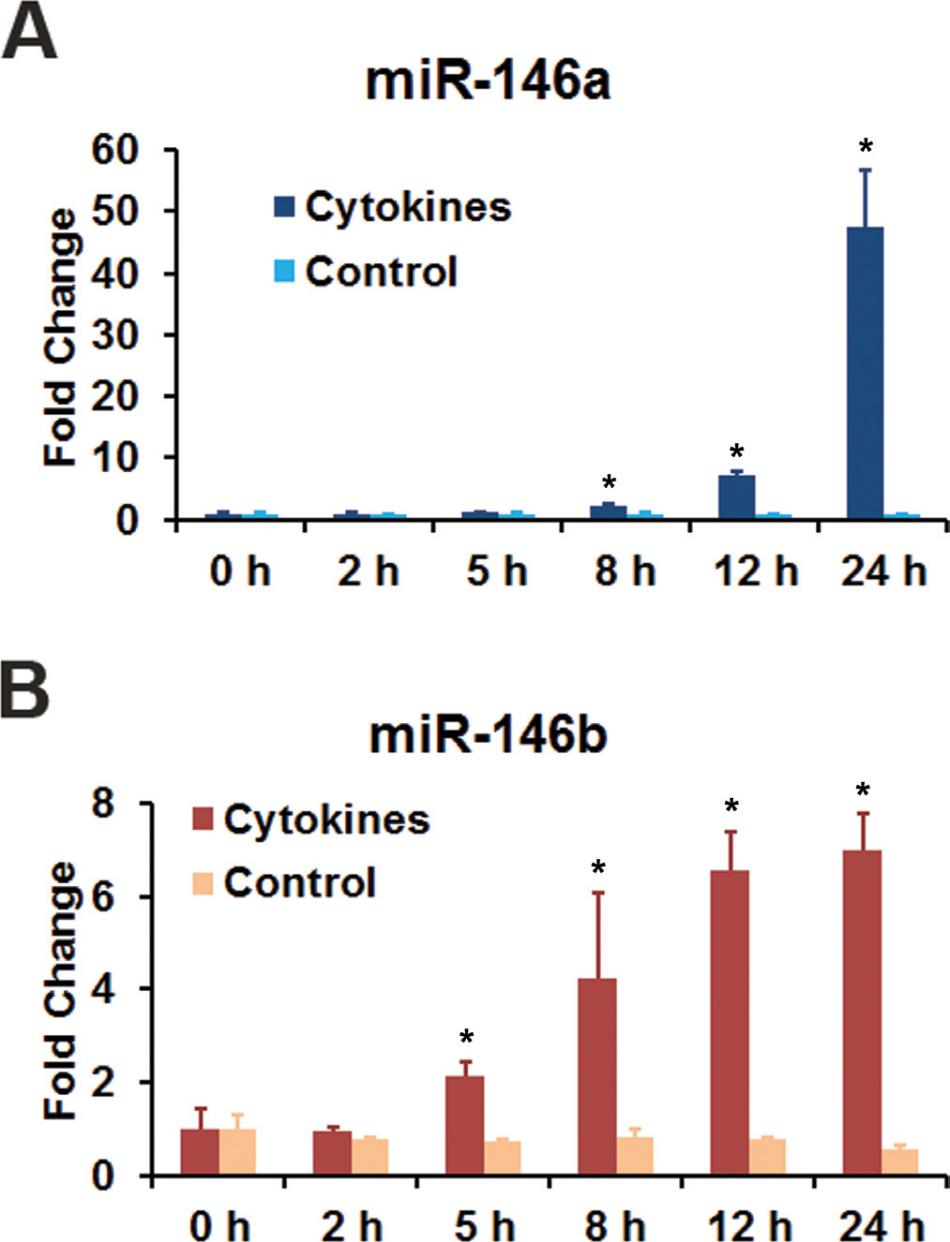
The increase in the expression of miR-146a and miR-146b-5p in human retinal pigment epithelial cells by the inflammatory cytokines was time dependent. The cells were treated with a combination of interferon (IFN)-γ (100 u/ml), tumor necrosis factor (TNF)-α (10 ng/ml), and interleukin (IL)-1β (10 ng/ml) for the indicated time intervals and the microRNA (miRNA) expression was analyzed by real-time PCR. *p<0.001 when compared to 0 h, n=4. The data shown are representative of three separate experiments.

**Figure 5:**
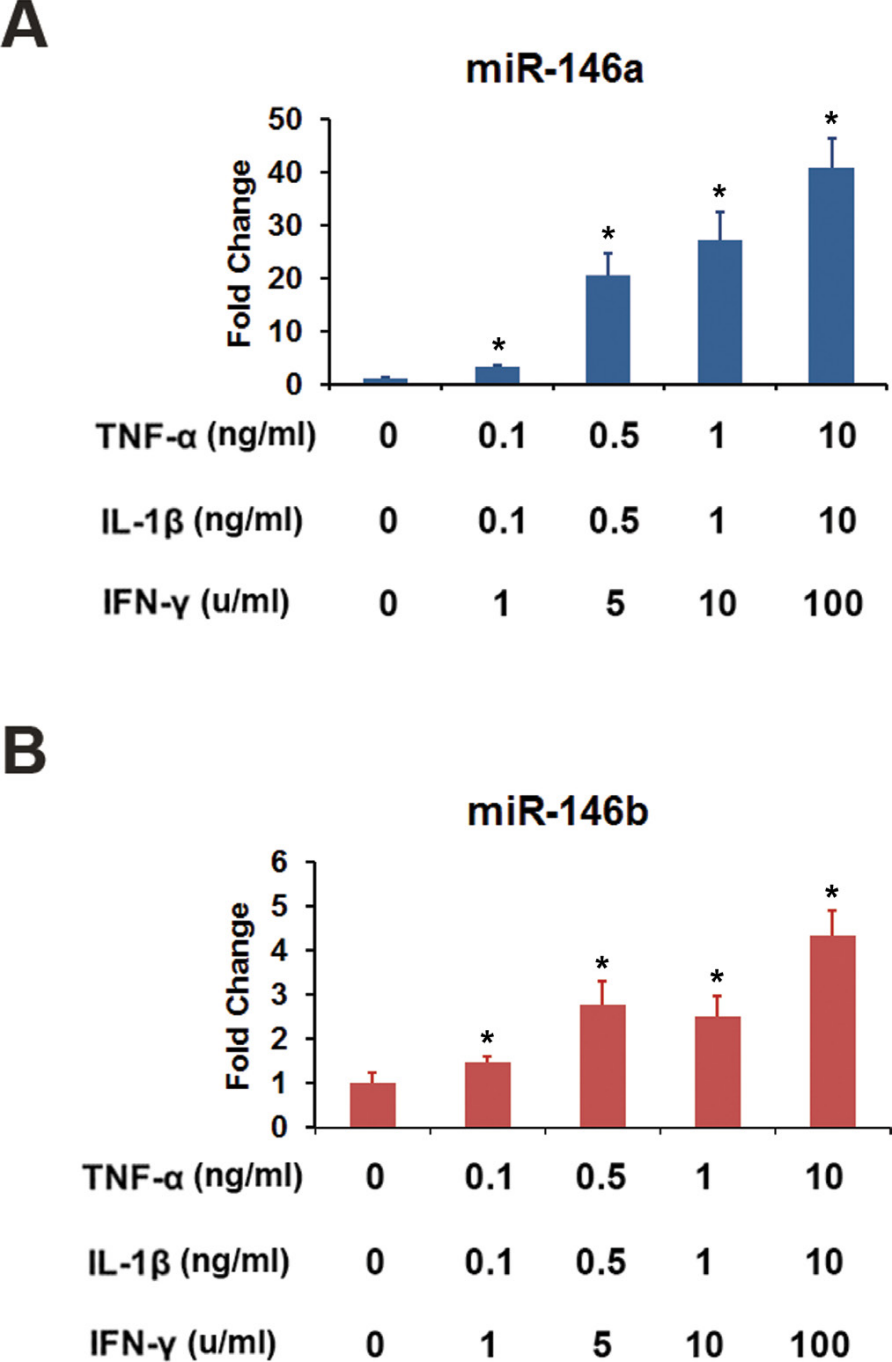
The increase in the expression of miR-146a and miR-146b-5p in human retinal pigment epithelial cells was dependent on the concentration of inflammatory cytokines. The microRNA (miRNA) expression was analyzed by real-time PCR after treating the cells with the indicated concentrations of interferon (IFN)-γ, tumor necrosis factor (TNF)-α, and interleukin (IL)-1β for 16 h. *p<0.002 when compared to control, n=4. The data from one of three similar experiments are shown here.

**Figure 6:**
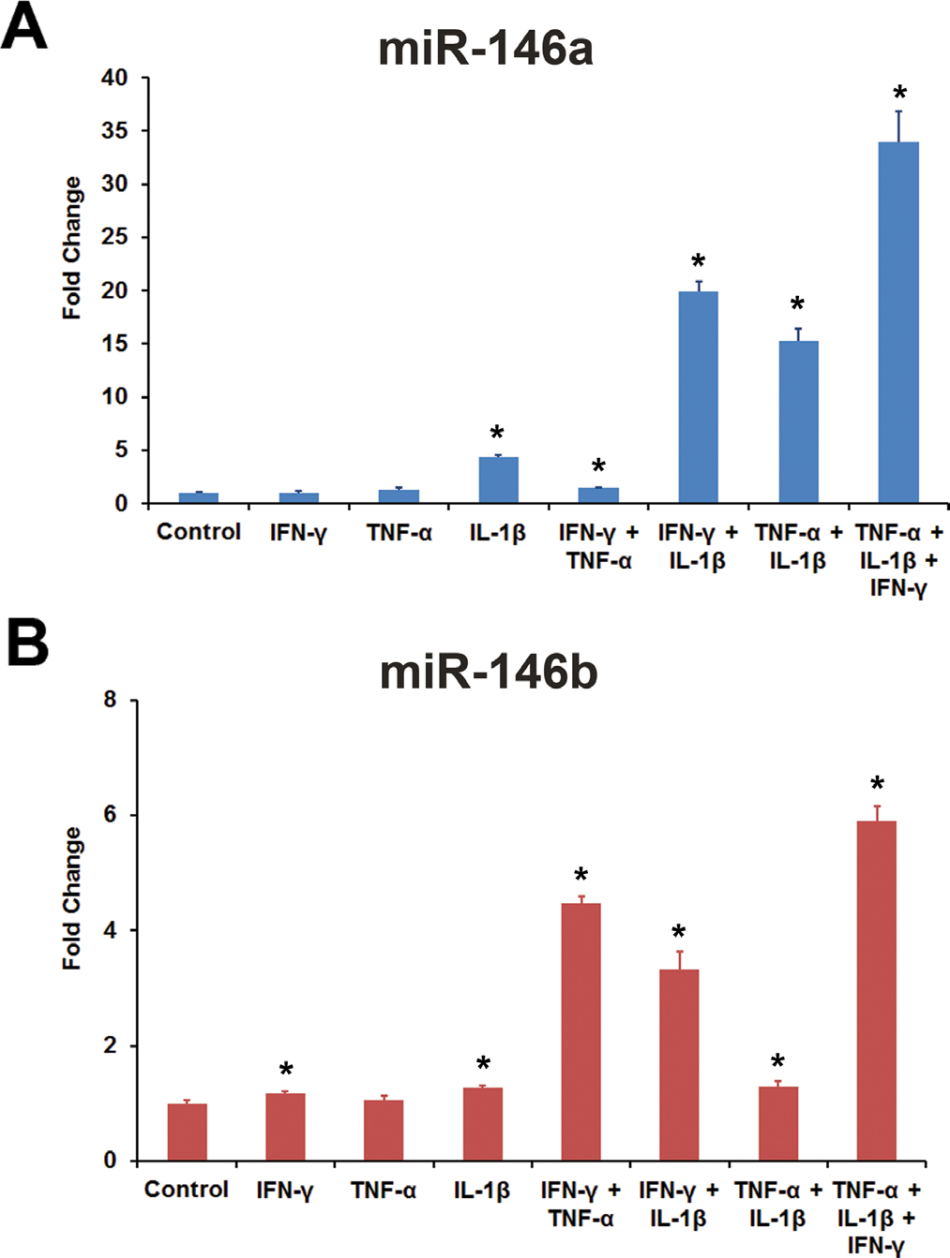
The expression of miR-146a and miR-146b-5p is differentially regulated by interferon-γ, tumor necrosis factor-α, and interleukin-1β. Human retinal pigment epithelial (HRPE) cells were treated with the indicated cytokine or cytokine combination for 16 h. The concentrations of interferon (IFN)-γ, tumor necrosis factor (TNF)-α, and interleukin (IL)-1β used were 100 u/ml, 10 ng/ml, and 10 ng/ml, respectively. The microRNA (miRNA) expression was analyzed by real-time PCR. A combination of all three cytokines elicited the highest increase in the expression of both miRNAs; however, miR-146a showed a dependency on IL-1β, while miR-146b-5p was dependent on IFN-γ. *p<0.001 when compared to control, n=4. The data shown are representative of three similar experiments.

The effect of proinflammatory cytokines on the expression of miR-146a and miR-146b-5p in HRPE cells was investigated further. Exposure of the cells to the cytokine mixture consisting of IFN-γ, TNF-α, and IL-1β resulted in a time-dependent increase in the expression of both miRNAs (Figure 4). However, the response of miR-146a was much slower than that of miR-146b-5p. The increase in expression of miR-146a and miR-146b-5p was also dependent on the concentration of cytokines (Figure 5). Noticeable increases in the expression of both miRNAs were observed even at lower concentrations of cytokines. We then investigated whether all three proinflammatory cytokines are required for inducing the expression of miR-146a and miR-146b-5p in HRPE cells (Figure 6). IL-1β was the most important component required for the induction of miR-146a. No appreciable induction was observed when it was omitted from the cytokine mixture. In addition, a noticeable increase in the expression of miR-146a was observed with IL-1β by itself. Both IFN-γ and TNF-α potentiated the effect of IL-1β, and the maximum increase in miR-146a expression was attained when all three cytokines were present. The increase in the expression of miR-146b-5p was dependent on the presence of IFN-γ. Induction was not observed when IFN-γ was omitted from the cytokine mixture. Both IL-1β and TNF-α were effective in inducing miR-146b-5p when paired with IFN-γ, and the maximum induction was detected when a combination of all three cytokines were used.

**Figure 7:**
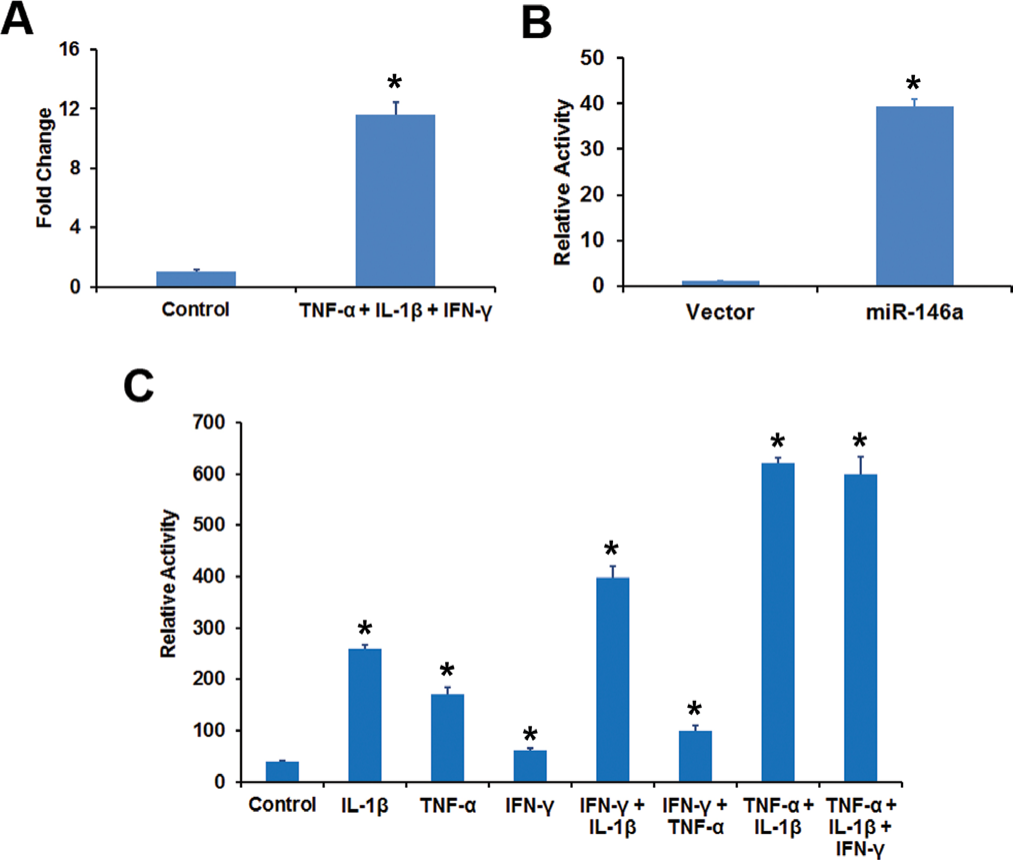
Proinflammatory cytokines increased *MIR146A* promoter activity in ARPE-19 cells. **A**: Inflammatory cytokines increased the expression of miR-146a in ARPE-19 cells. The cells were treated with a mixture of interferon (IFN)-γ (100 u/ml), tumor necrosis factor (TNF)-α (10 ng/ml), and interleukin (IL)-1β (10 ng/ml) and the miR-146a expression analyzed by real-time PCR. *p<0.001 when compared to control, n=4. **B**: *MIR146A* promoter activity in ARPE-19 cells. The cells were transfected with either a *MIR146A* promoter-luciferase construct or the vector and the luciferase activity was estimated as a measure of promoter activity. *p<0.001 when compared to vector, n=3. **C**: Inflammatory cytokines increased the *MIR146A* promoter activity in ARPE-19 cells. The cells were first transfected with the *MIR146A* promoter-luciferase construct and then treated with the indicated cytokines (IFN-γ, 100 u/ml; TNF-α, 10 ng/ml; or IL-1β, 10 ng/ml) or their combination for 24 h. The promoter activity was estimated by measuring the luciferase activity. *p<0.01 when compared to control, n=3. The data shown are representative of three similar experiments.

**Figure 8:**
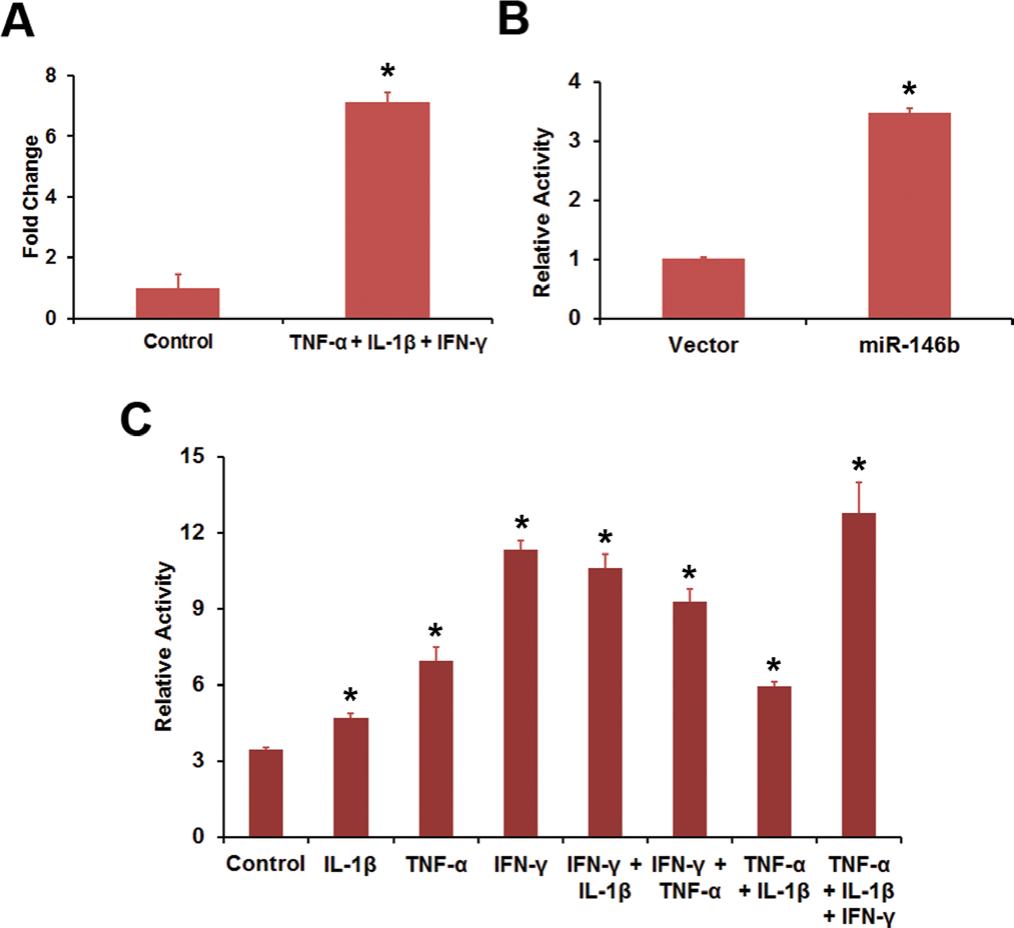
Proinflammatory cytokines increased *MIR146B* promoter activity in ARPE-19 cells. **A**: Inflammatory cytokines increased the expression of miR-146b-5p in ARPE-19 cells. The cells were treated with a mixture of interferon (IFN)-γ (100 u/ml), tumor necrosis factor (TNF)-α (10 ng/ml), and interleukin (IL)-1β (10 ng/ml) for 16 h and the miR-146b-5p expression analyzed by real-time PCR. *p<0.001 when compared to control, n=4. **B**: *MIR146B* promoter activity in ARPE-19 cells. The cells were transfected with either a *MIR146B* promoter-luciferase construct or the vector and the luciferase activity was estimated as a measure of promoter activity. *p<0.001 when compared to vector, n=3. **C**: Inflammatory cytokines increased the *MIR146B* promoter activity in ARPE-19 cells. The cells were first transfected with the *MIR146B* promoter-luciferase construct and then treated with the indicated cytokines (IFN-γ, 100 u/ml; TNF-α, 10 ng/ml; or IL-1β, 10 ng/ml) or their combination for 24 h. The promoter activity was estimated by measuring the luciferase activity. *p<0.01 when compared to control, n=3. The data shown are representative of three similar experiments.

We employed the RPE-derived cell line, ARPE-19 [31], to analyze the effect of IFN-γ, TNF-α, and IL-1β on the promoter activity of the genes encoding miR-146a and miR-146b-5p. These cells responded to the proinflammatory cytokines by increasing the expression of miR-146a (Figure 7A). An *MIR146A* promoter-luciferase construct exhibited promoter activity when compared to vector alone (Figure 7B). The promoter activity was increased by proinflammatory cytokine treatment (Figure 7C). IL-1β yielded the best response when the cytokines were tested individually. Combining IL-1β with IFN-γ, TNF-α, or both further increased the promoter activity. However, the omission of IL-1β from the cytokine mixture resulted in low *MIR146A* promoter activity. Thus, IL-1β appears to be the critical proinflammatory cytokine regulating the *MIR146A* promoter activity. The expression of miR-146b-5p was also increased in ARPE-19 cells by the cytokine mixture (Figure 8A). An *MIR146B* promoter-luciferase construct showed promoter activity in transfected ARPE-19 cells (Figure 8B). The promoter activity increased when cells were treated with IFN-γ, TNF-α, or IL-1β (Figure 8C). The best response was observed with IFN-γ and combining it with other cytokines produced the maximum effect. Thus, IFN-γ is the most important proinflammatory cytokine for regulating *MIR146B* promoter activity.

**Figure 9:**
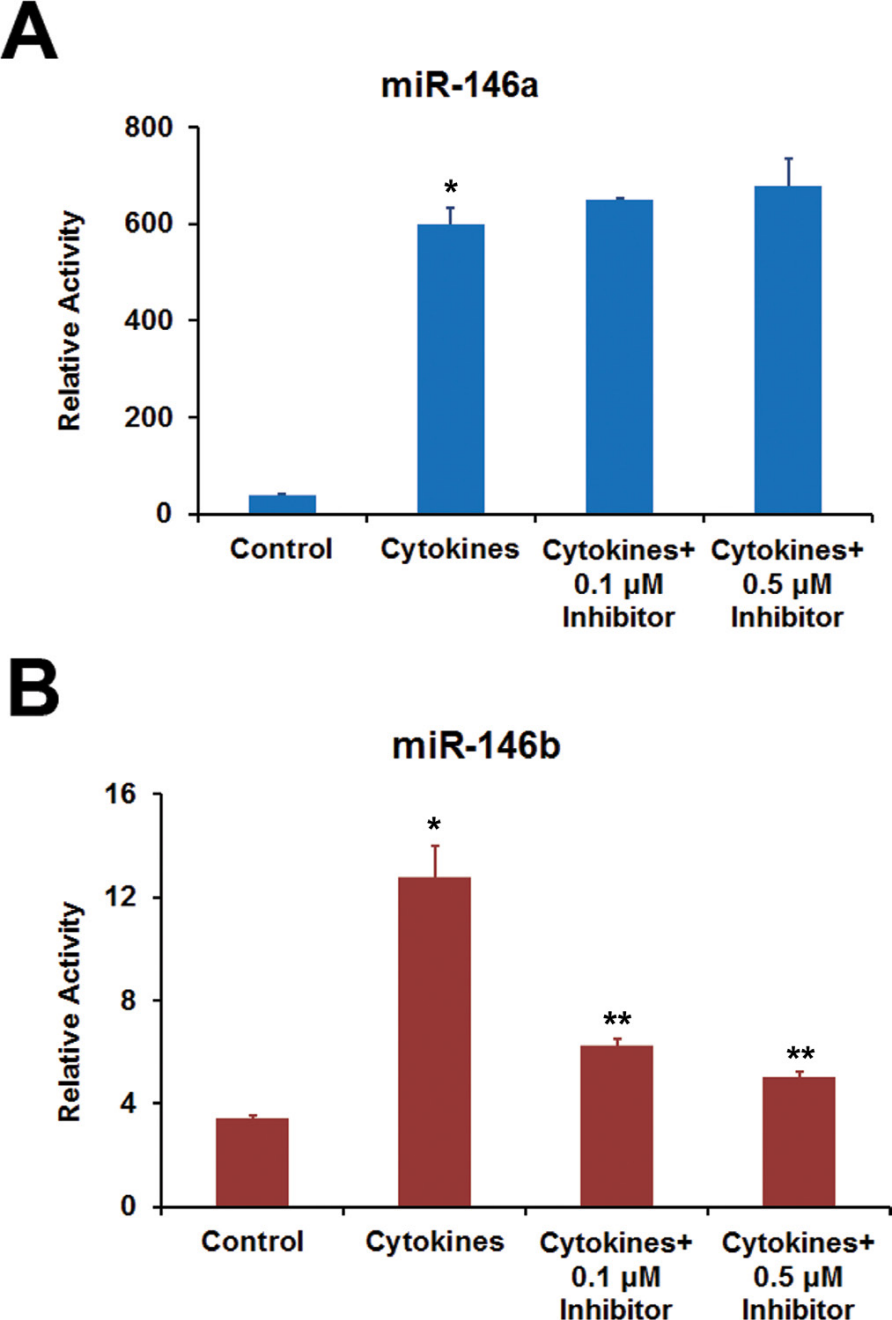
The activation of *MIR146A* promoter by proinflammatory cytokines in ARPE-19 cells was not affected by JAK inhibitor 1, while that of the *MIR146B* promoter was effectively blocked. The cells were transfected with promoter-luciferase constructs of *MIR146A* or *MIR146B*, and treated with a mixture of interferon (IFN)-γ (100 u/ml), tumor necrosis factor (TNF)-α (10 ng/ml), and interleukin (IL)-1β (10 ng/ml) for 16 h in the presence or absence of JAK inhibitor 1. The promoter activity was estimated by measuring the luciferase activity. *p<0.01 when compared to control, n=3; **p<0.01 when compared to cytokines, n=3. The data shown are representative of three similar experiments.

The role of IFN-γ in regulating *MIR146A* and *MIR146B* promoter activity was investigated using JAK inhibitor 1, a known blocker of IFN-γ signaling via the JAK/STAT pathway (Figure 9). The increase in *MIR146A* promoter activity by the cytokine mixture in ARPE-19 cells was not affected by JAK inhibitor 1. However, the increase in *MIR146B* promoter activity resulting from the cytokine treatment was effectively blocked by JAK inhibitor 1. Thus, IFN-γ appears to regulate *MIR146B* promoter activity via the JAK/STAT signaling pathway.

**Figure 10:**
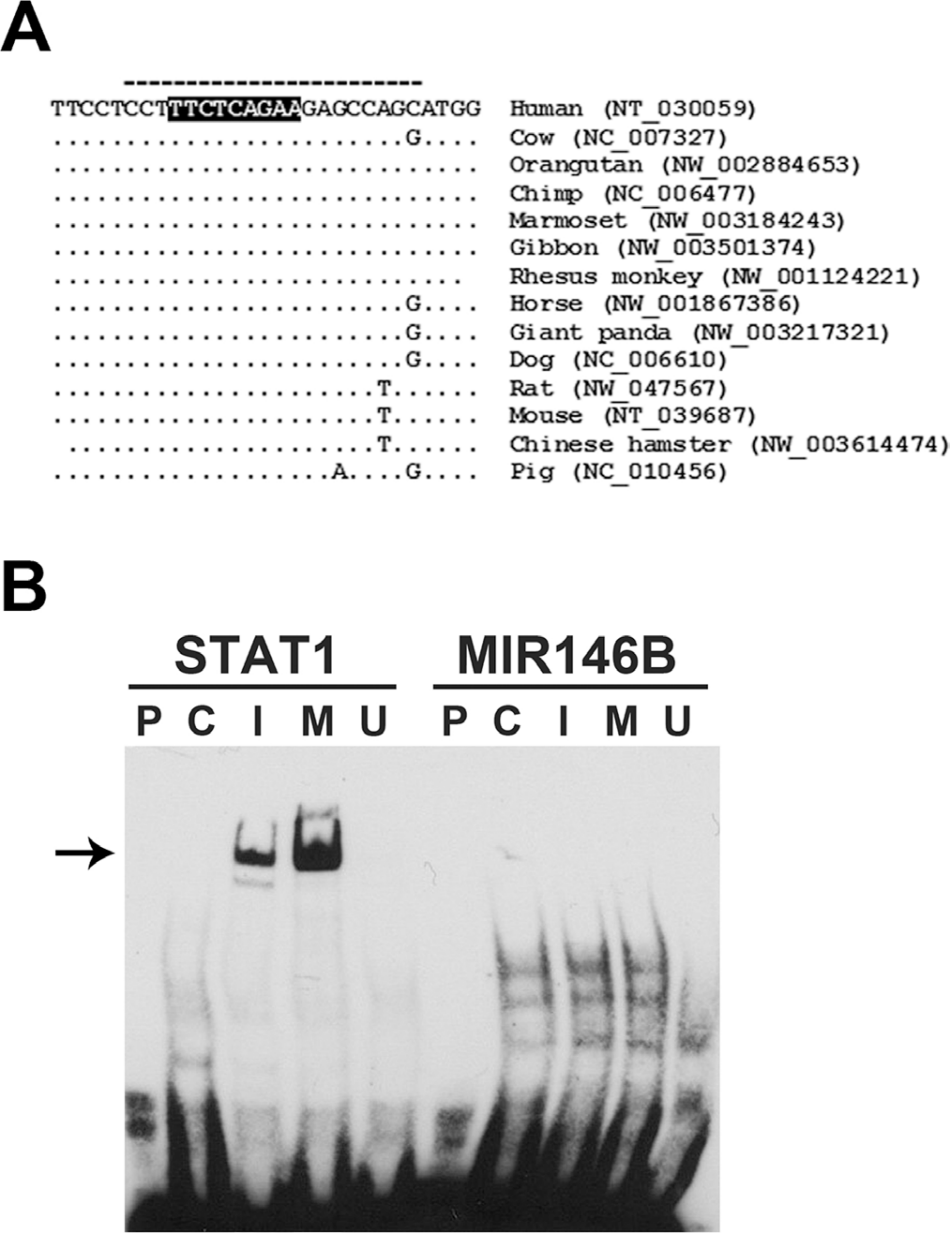
Electrophoretic mobility shift assay of STAT1 activation. **A**: A putative STAT-binding element (shown in dark background) that is evolutionarily conserved in mammalian species was detected in the promoter region of *MIR146B*. The dashed line indicates the sequence of the oligonucleotide probe used for the electrophoretic mobility shift assay. **B**: Electrophoretic mobility shift assay shows that the putative *MIR146B* STAT-binding element lacks the ability to bind STAT1. The assay was performed with biotin-labeled oligonucleotides containing either a known STAT1-binding site (shown as STAT1, left side of the blot in the figure) or the putative STAT-binding element of *MIR146B* (shown as MIR146B, right side of the blot in the figure) and using nuclear extracts from human retinal pigment epithelial (HRPE) cells. The arrow identifies the STAT1 protein binding. Lane P, probe alone; lane C, extract from control cells; lane I, extract from cells treated with interferon (IFN)-γ; lane M, extract from cells treated with a mixture of IFN-γ, tumor necrosis factor (TNF)-α, and interleukin (IL)-1β; lane U, a 50-fold excess of corresponding unlabeled oligonucleotide and extract from cells treated with a mixture of IFN-γ, TNF-α, and IL-1β. The data from one of the two similar experiments are shown.

We analyzed the *MIR146B* promoter sequence that was used for making the promoter-reporter construct for the presence of potential STAT1 binding sites. A potential STAT-binding element that is conserved in many mammalian species was detected in the human *MIR146B* promoter sequence (Figure 10 A). The ability of oligonucleotides containing this STAT-binding element to bind STAT1 transcription factor was studied by electrophoretic mobility shift assay using nuclear extracts from HRPE cells (Figure 10B). Studies with a control oligonucleotide containing known STAT1 binding sequence clearly have shown that the STAT1-binding activity was highly induced in HRPE cells by IFN-γ either alone or in combination with IL-1β and TNF-α. However, no STAT1-binding activity was detected when the oligonucleotide containing potential STAT-binding element of *MIR146B* was used. Therefore, the highly conserved potential binding site that we identified may not be involved in the activation of *MIR146B* promoter by IFN-γ.

**Figure 11:**
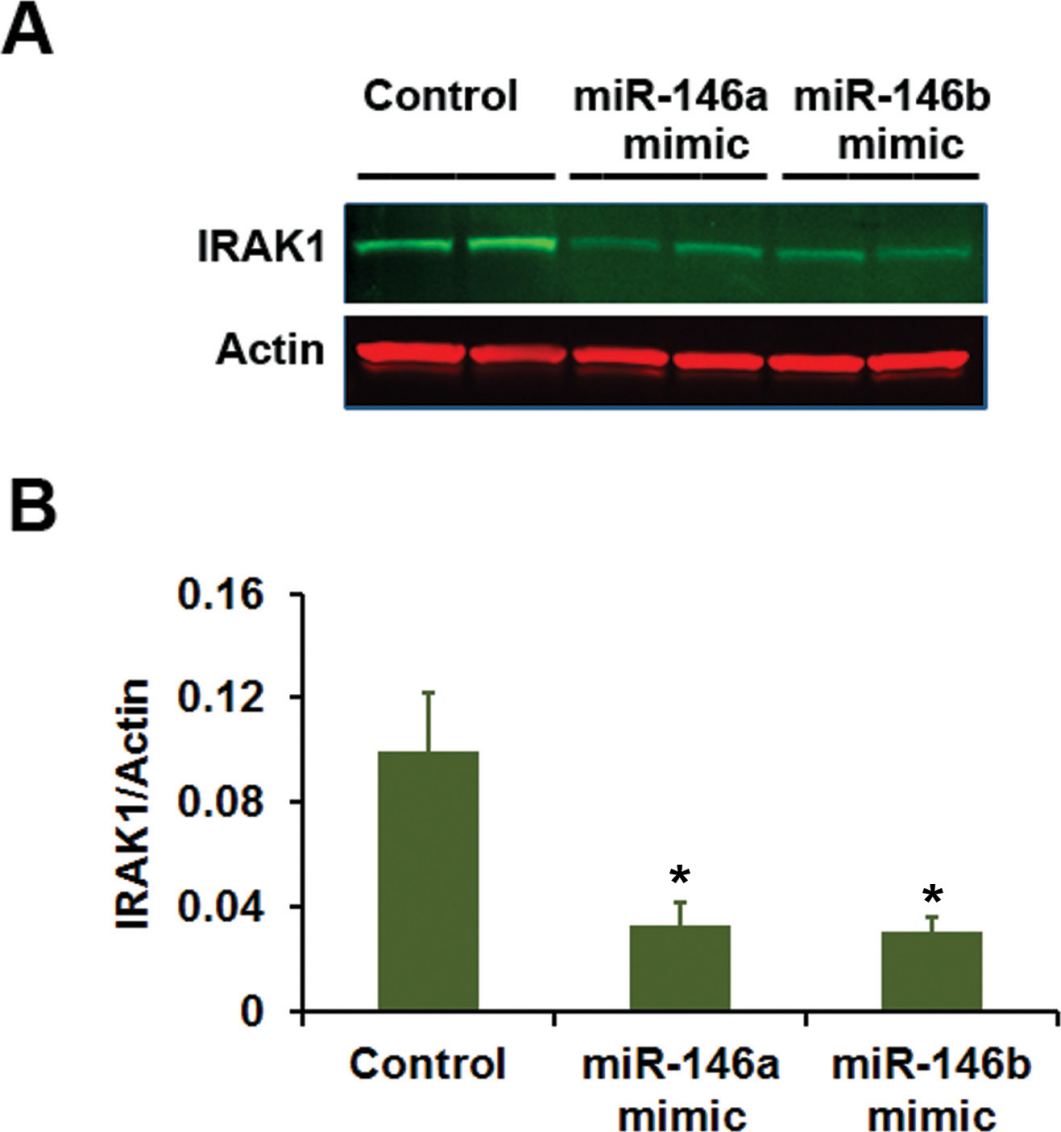
The expression of IRAK1 protein in ARPE-19 cells is decreased by miR-146a and miR-146b-5p mimics. The cells were transiently transfected with microRNA (miRNA) mimics for 72 h. **A**: Western immunoblot analysis of IRAK1 expression. Cells were treated with control miRNA mimic (random sequence), miR-146a mimic, or miR-146b-5p mimic. Actin was used as the loading control. A representative blot from three similar experiments is shown. **B**: Histogram showing IRAK1 expression. The IRAK1 band intensity was normalized with corresponding actin band intensity; *p<0.01 when compared to control, n=6.

IRAK1 is reported to be a target for translational repression by miR-146 [17]. We tested the ability of miR-146a, as well as that of miR-146b-5p, to regulate the expression of IRAK1 in ARPE-19 cells. Western blot analysis showed that the cells transfected with miR-146a mimic and miR-146b-5p mimic expressed decreased amounts of IRAK1 protein when compared to those transfected with control miRNA with unrelated sequence (Figure 11). Thus, both miR-146a and miR-146b-5p have the ability to suppress the expression of IRAK1 protein in RPE cells in culture. We made an attempt to study the effect of miR-146a and miR-146b mimics on the production of chemokines and cytokines by ARPE-19 cells. The cells were treated with the miRNA mimics and the chemokine and cytokine concentrations in the culture medium were analyzed by enzyme-linked immunosorbent assay. Our results show that the CCL2 concentration was decreased from 4.6 and 4.7 ng/ml to 2.9 and 2.9 ng/ml following miR-146a mimic treatment, and to 3.0 and 3.7 ng/ml following miR-146b-5p mimic treatment. The IL-8 concentration decreased from 1.0 and 1.0 ng/ml to 0.7 and 0.8 ng/ml following miR-146a mimic treatment and to 0.7 and 0.8 ng/ml following miR-146b-5p mimic treatment. Therefore, the decrease in IRAK1 protein in ARPE-19 cells in response to treatment with miR-146a mimic or miR-146b-5p mimic is associated with noticeable decreases in the production of CCL2 and IL-8 by these cells.

## Discussion

Targeting of RPE by IFN-γ, TNF-α, and IL-1β, proinflammatory cytokines secreted by infiltrating lymphocytes and macrophages during ocular inflammation, could be an important factor in the pathogenesis of AMD [6]. When secreted into the posterior compartment of the eye, these inflammatory mediators can potentially interact with RPE and seriously impair its critical functions [2,3]. Proinflammatory cytokines such as IL-1β and TNF-α can signal RPE to produce chemokines and cytokines via the NF-κB pathway [10,11]. NF-κB activation, if not regulated, can lead to an uncontrolled inflammatory response detrimental to RPE function. Our study shows for the first time that RPE cells in culture (HRPE and ARPE-19) do express miR-146a and miR-146b-5p, two closely related miR-146 family members known to negatively regulate cytokine signaling via the NF-κB signaling pathway [17]. In addition, the expression of both of these miRNAs increased several fold in RPE cells following treatment with a combination of IFN-γ, TNF-α, and IL-1β. In our results, a significant increase in miR-146a and miR-146b expression was observed at TNF-α, IL-1β, and IFN-γ concentrations of 0.10 ng/ml, 0.10 ng/ml and 1 unit/ml (~0.05 ng/ml), respectively. It is not known whether these concentrations are pathophysiologically relevant. Reports on intraocular concentrations of these cytokines under in vivo conditions are limited, except in the study of Okada et al. [32]. They observed that the intraocular concentrations of TNF-α and IFN-γ reached 0.0835±0.0634 ng/ml and 0.331±0.124 ng/ml, respectively, in rats with experimental autoimmune uveoretinitis. We detected low-level constitutive expression of both miR-146a and miR-146b-5p in RPE cells. Interestingly, the expression of both these miRNAs is undetectable in primary B lymphocytes, although it is highly increased following Epstein-Barr virus infection [21].

MiR-146 is implicated in a variety of cellular functions, including oncogenesis, infection, immune response, and inflammation. The induction of miR-146 in response to inflammatory stimuli has been reported in murine macrophages, human acute monocytic leukemia cell line THP-1, and human synovial fibroblasts [17,25,33]. This miRNA is thought to play a role during the pathogenesis of rheumatoid arthritis, an inflammatory disease [25]. MiR-146a and miR-146b-5p are known to negatively regulate inflammatory process due to their ability to suppress the NF-κB signaling pathway by targeting IRAK1 and TRAF6 for translational repression [17]. Our study has shown that IRAK1 could be a potential target for miR-146a and miR-146b-5p in RPE cells in culture. The expression of IRAK1 protein is greatly decreased in cells after transfection with mimics for either of these miRNAs. Thus, due to its inherent ability to translationally repress IRAK1 expression, miR-146 could also suppress the ocular inflammatory process and play a beneficial role in the pathogenesis of AMD. According to Lukiw et al., however, due to its ability to translationally repress the expression of complement factor H (CFH), miR-146a could also have a detrimental role in AMD pathogenesis [34]. These researchers’ hypothesis is supported by the increased presence of miR-146a observed in the retina of AMD patients in comparison to age-matched controls, and the association of CFH mutation with this disease [34,35]. Further studies are needed to delineate the opposing roles of IRAK1 and CFH in the etiology of AMD. The ability of miR-146a to target the expression of CFH in RPE cells remains to be elucidated. IRAK2, IL-8, RANTES (CCL5), and CCL8 are known to be targeted by this miRNA during the inflammatory response [19,33,36]. Interestingly, we have observed a decrease in the production of IL-8 and CCL2 by RPE cells in culture following treatment with either miR-146a mimic or miR-146b-5p mimic as mentioned in Results.

Recent studies have revealed that microRNAs could be major players in RPE pathophysiology. Several miRNAs are expressed in RPE and retina [37,38]. Wang et al. reported that miR-204 and miR-211 play critical roles in maintaining epithelial physiology and barrier function of RPE [38]. Dedifferentiation of cultured fetal HRPE cells is a consequence of the decreased expression of miR-204 and miR-211 resulting from the downregulation of microphthalmia-associated transcription factor [39]. We have shown that a large number of miRNAs are expressed in the human retinal pigment epithelial cell line, ARPE-19 [40]. The expression of miR-9 in ARPE-19 cells is increased during apoptosis induced by N-(4-hydroxyphenyl)retinamide [40]. We have reported previously that HRPE cells respond to TNF-α, IL-1β, and IFN-γ by increasing the expression of miR-155 [29]. The present study shows that the proinflammatory cytokines can induce the expression of miR-146a and miR-146b-5p in addition to miR-155. It is interesting to note that miR-155 may also act as a negative regulator of the inflammatory process due to its ability to regulate the expression of I-kappa-B kinase epsilon (IKKε), CCAAT/enhancer-binding protein β (CEBPB), and src homology-2 domain-containing inositol-5’-phosphatase 1 (SHIP1) by translational repression [41-43].

The effect of TNF-α, IL-1β, and IFN-γ on the induction of miR-146a expression in HRPE cells was synergistic, with maximum induction being observed when all three cytokines were combined. However, this induction was mostly dependent on IL-1β and least on IFN-γ. This tendency was also seen for the promoter activity. IL-1β could elicit its effect in RPE cells by activating the NF-κB signaling pathway to induce miR-146a induction, as clearly established in other systems [17,22,44].

Unlike miR-146a, the regulation of miR-146b-5p expression has not been investigated in detail except in a report by Perry et al. that found that MEK-1/2 and JNK-1/2 pathways regulate its induction in alveolar epithelial cells by IL-1β [44]. In our study using cultured RPE cells, the increased expression of miR-146b-5p in RPE cells by proinflammatory cytokines showed a dependency on IFN-γ rather than IL-1β. In addition, its promoter activity was increased considerably by IFN-γ, and not by IL-1β. The increase in the promoter activity was effectively blocked by an inhibitor of JAK/STAT pathway, supporting the role of IFN-γ. Thus, our study has shown for the first time that miR-146b-5p expression is under the control of IFN-γ, indicating that it may have an important role in the inflammatory process. The role of miR-146b-5p in inflammation has been recently questioned because its expression has not been detected in adult lymphoid or myeloid cells [45]. The possibility that its detection using hybridization-based techniques can be compromised by the potential cross-hybridization of miR-146a and miR-146b-5p probes has also been raised. The miR-146b-5p expression in RPE cells is clearly distinct from that of miR-146a. In our case, the expression of miR-146b-5p in control RPE cells was much higher than miR-146a when analyzed by real-time PCR. In addition, we were able to detect the expression of pre-miR-146b in RNA fraction obtained from these cells (data not shown). We have also noted striking temporal differences in the expression of miR-146b-5p and miR-146a in RPE cells. The time response of miR-146b-5p induction by proinflammatory cytokines was considerably faster than that of miR-146a induction. The induction of miR-146b-5p was IFN-γ dependent while that of miR-146a expression was IL-1β-dependent.

Although IFN-γ is known to elicit its action via JAK/STAT signaling pathway, the direct involvement of this pathway in regulating miR-146b-5p expression needs to be revealed. We analyzed the *MIR146B* gene promoter region for the presence of STAT1 binding elements to study the role of JAK/STAT signaling pathway in mediating the induction of miR-146b-5p by inflammatory cytokines in HRPE cells. We detected a putative binding sequence based on the DNA binding specificity attributed to STAT1 [46,47]. However, oligonucleotides containing this element failed to bind protein(s) present in the nuclear extracts of HRPE cells in our electrophoretic mobility shift assays. Therefore, the increase in the expression of miR-146b-5p in HRPE cells on exposure to inflammatory cytokines was not associated with STAT1 binding to this particular binding site. It is possible that potential STAT1 binding sites could be present further upstream of the sequence region that we analyzed. Thus, our studies indicate for the first time that IFN-γ could regulate miR-146b-5p expression via the JAK/STAT signaling pathway; however, the role of STAT1 in this process remains to be elucidated. Moreover, it is not known whether STAT1 is a target for regulation by miR-146b-5p. Interestingly, Lu et al. [48] have reported that miR-146a acts as a repressor of Stat1 activation in mouse regulatory T (Treg) cells. Therefore, the IFN-γ-mediated induction of miR-146b-5p could be seen as a potential mechanism that can negatively regulate the JAK/STAT signaling pathway.

**Figure 12:**
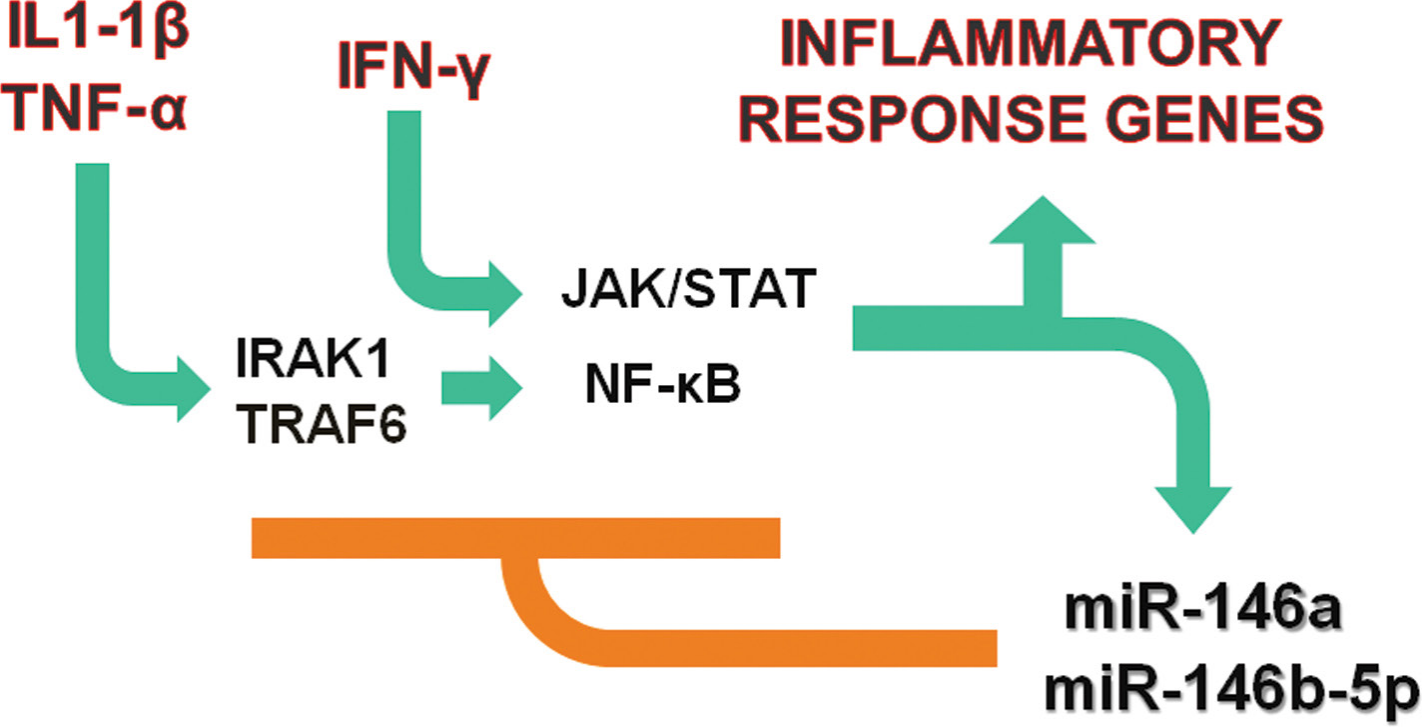
MicroRNA-146a and microRNA-146b-5p can potentially negatively regulate the inflammatory response of the retinal pigment epithelium. The schematic diagram shows that the retinal pigment epithelial (RPE) cells respond to the proinflammatory cytokines interleukin-1β (IL-1β), tumor necrosis factor-α (TNF-α), and interferon-γ (IFN-γ) by increasing the expression of inflammatory response genes. IL-1β and TNF-α regulate the gene expression by binding to specific cell surface receptors and thereby activating the nuclear factor (NF)-κB signaling pathway. Interleukin-1-receptor-associated kinase-1 (IRAK1) and TNF receptor associated factor 6 (TRAF6) are known modulators of the NF-κB pathway. IFN-γ alters gene expression by interacting with its cell surface receptors and then activating the Janus kinase (JAK)/signal transducer and activator of transcription (STAT) signal transduction pathway. The activation of NF-κB and JAK/STAT pathways by the proinflammatory cytokines can also cause an increase in the expression of miR-146a and miR-146b-5p in the RPE cells. Both miRNAs can then act as negative feedback regulators of the inflammatory response by targeting IRAK-1 or other modulators of the NF-κB or JAK/STAT signal transduction pathways for translational repression.

Our results (Figure 2, Figure 3, Figure 4 and Figure 5) clearly show that the inflammatory cytokines (a combination of TNF-α, IL-1β, and IFN-γ) markedly increased the expression of miR-146a and miR-146b-5p in HRPE cells. The induction of miR-146a was more dependent on IL-1β, while that of miR-146b-5p was more dependent on IFN-γ. We have also provided evidence that IFN-γ and the JAK/STAT signaling pathway could be involved in the regulation of miR-146b-5p expression. Both miRNAs could modulate the response of the RPE cells to inflammatory stimuli by potentially targeting IRAK1 (Figure 12). These microRNAs have the potential to serve as therapeutic targets for arresting retinal degenerative diseases resulting from unregulated RPE inflammatory response.
